# Supplementary material for: Sensory computations in the cuneate nucleus of macaques
Source: Proc Natl Acad Sci U S A. 2021 Dec 1;118(49):e2115772118. doi: 10.1073/pnas.2115772118 (PMC8670430; doi:10.1073/pnas.2115772118)
Supplement: Supplementary File [file pnas.2115772118.sapp.pdf]

Supporting Information

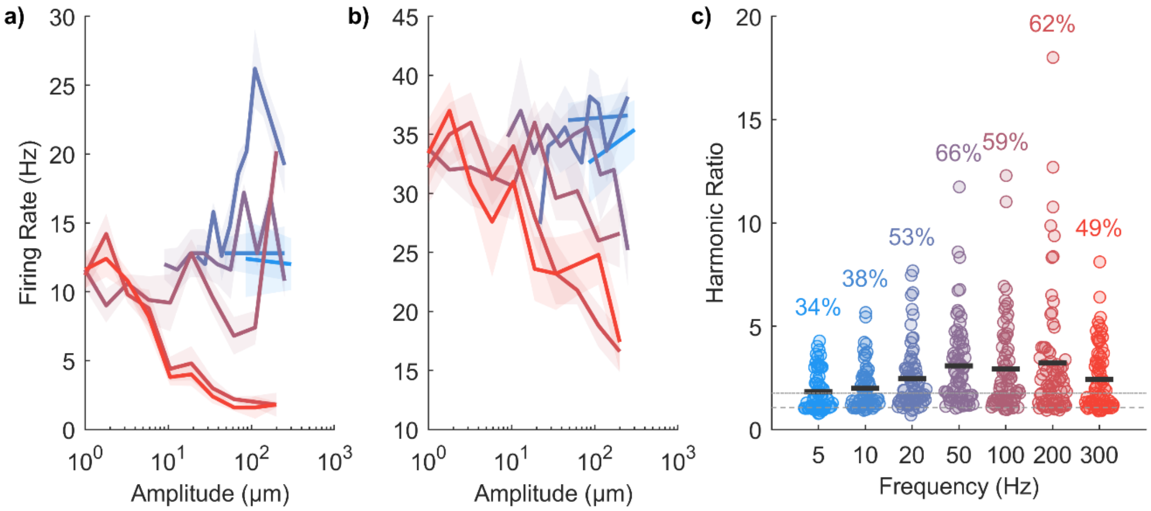

Supplementary Figure 1: **Frequency characteristics of CN neurons.** a & b| Example rate-intensity plots for two CN neurons. The neuron in panel *a* is sometimes excited, sometimes inhibited by skin vibrations depending on their frequency while the neuron in panel *b* exhibits only suppression. c| The proportion of neurons that are significantly tuned to each frequency. The dashed line indicates the mean harmonic ratio of a Poisson neuron (~1), and the dotted line above is the value 3 standard deviations above the mean.

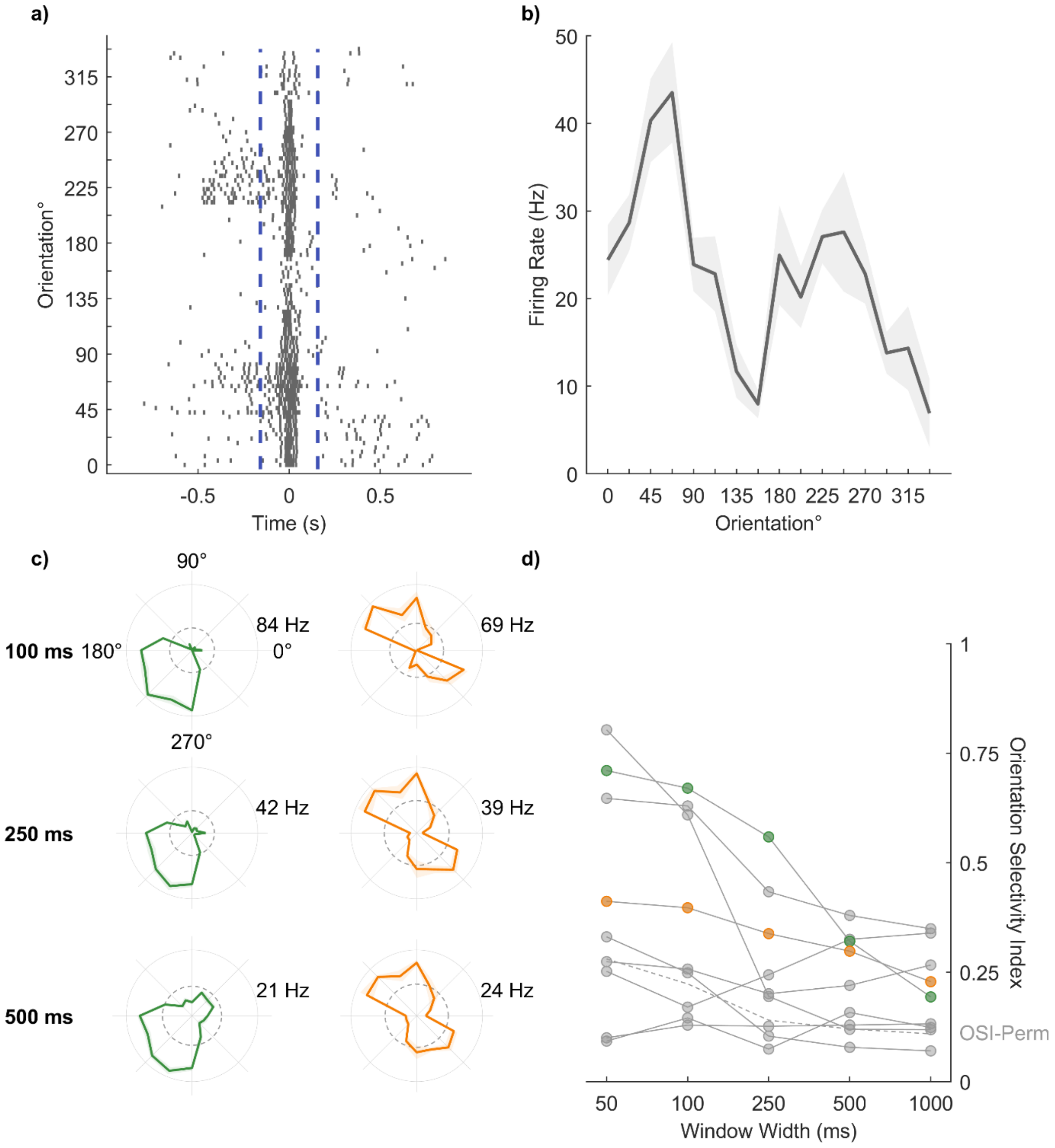

Supplementary Figure 2: **Orientation tuning is stable with respect to window size.** a| Raster plot of the responses of an example CN neuron to edges at each orientation aligned to the peak response. b| Tuning curve for the neuron shown in panel a. c| Polar plots for 2 example neurons computed from the responses averaged over 3 time windows. d| Vector strength for all neurons computed over different windows. Example neurons from panel c are highlighted in the corresponding color. OSI-Perm indicates the orientation index averaged across neurons when the firing rates are shuffled across trials.

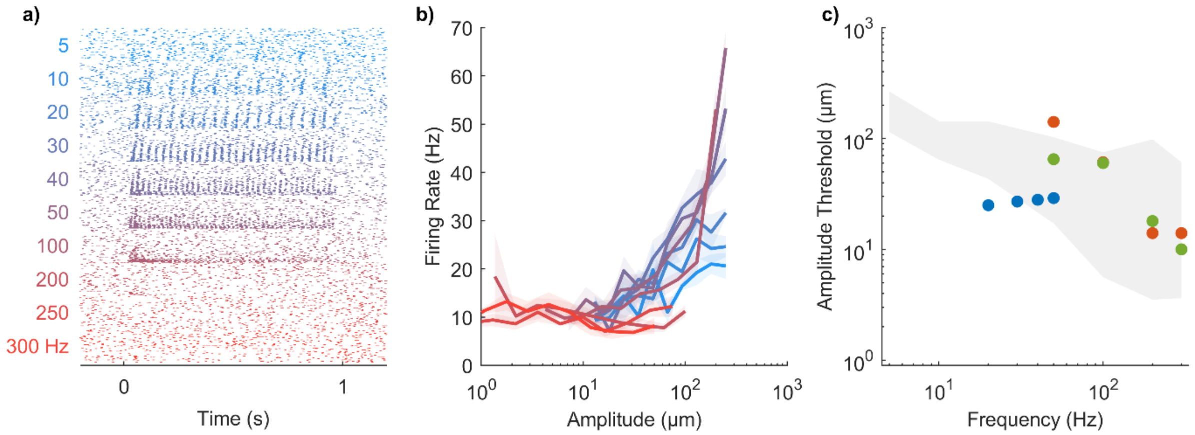

Supplementary Figure 3: **Vibrotactile responses are minimally altered by anesthesia.** a| Example raster and b| rate-intensity function for a neuron recorded from an awake monkey with a chronic array. c| Thresholds derived from the responses of three neurons measured in awake monkeys were similar to their counterparts measured under anesthesia. For two neurons (denoted by green and orange dots), the vibratory stimuli were delivered using a different vibratory stimulator (mini shaker, 4810, Bruel and Kjaer, Naerum, Denmark). For all three neurons, the contactor was smaller (1 mm) than that used in the acute experiments. The gray outline shows the range of thresholds observed for anesthetized monkeys while dots indicate the thresholds for the units monitored in awake monkeys.
